# STAT3 antisense oligonucleotide AZD9150 in a subset of patients with heavily pretreated lymphoma: results of a phase 1b trial.

**DOI:** 10.1186/s40425-018-0436-5

**Published:** 2018-11-16

**Authors:** Matthew J. Reilley, Patricia McCoon, Carl Cook, Paul Lyne, Razelle Kurzrock, Youngsoo Kim, Richard Woessner, Anas Younes, John Nemunaitis, Nathan Fowler, Michael Curran, Qinying Liu, Tianyuan Zhou, Joanna Schmidt, Minji Jo, Samantha J. Lee, Mason Yamashita, Steven G. Hughes, Luis Fayad, Sarina Piha-Paul, Murali V. P. Nadella, Xiaokun Xiao, Jeff Hsu, Alexey Revenko, Brett P. Monia, A. Robert MacLeod, David S. Hong

**Affiliations:** 1 Division of Hematology/Oncology, University of Virginia Health System, Charlottesville, VA USA; 2 Oncology, IMED Biotech Unit, AstraZeneca Pharmaceuticals, Waltham, MA USA; 3 UC San Diego Moores Cancer Center, La Jolla, CA USA; 4 Department of Antisense Drug Discovery, Ionis Pharmaceuticals, Inc., Carlsbad, CA USA; 5 Memorial Sloan Kettering Cancer Center, New York, NY USA; 6 Mary Crowley Cancer Research Center, Dallas, TX USA; 7 Department of Investigational Cancer Therapeutics, The University of Texas MD Anderson Cancer Center, 1515 Holcombe Blvd., Unit 0455, Houston, TX 77030 USA; 8 Drug Safety and Metabolism, IMED Biotech Unit, AstraZeneca Pharmaceuticals, Waltham, MA USA

**Keywords:** STAT3, Anti-sense oligonucleotide, Diffuse large B-cell lymphoma, DLBCL, Clinical trial, Immunotherapy, Lymphoma, JAK-STAT

## Abstract

**Background:**

The Janus kinase (JAK) and signal transduction and activation of transcription (STAT) signaling pathway is an attractive target in multiple cancers. Activation of the JAK-STAT pathway is important in both tumorigenesis and activation of immune responses. In diffuse large B-cell lymphoma (DLBCL), the transcription factor STAT3 has been associated with aggressive disease phenotype and worse overall survival. While multiple therapies inhibit upstream signaling, there has been limited success in selectively targeting STAT3 in patients. Antisense oligonucleotides (ASOs) represent a compelling therapeutic approach to target difficult to drug proteins such as STAT3 through of mRNA targeting. We report the evaluation of a next generation STAT3 ASO (AZD9150) in a non-Hodgkin’s lymphoma population, primarily consisting of patients with DLBCL.

**Methods:**

Patients with relapsed or treatment refractory lymphoma were enrolled in this expansion cohort. AZD9150 was administered at 2 mg/kg and the 3 mg/kg (MTD determined by escalation cohort) dose levels with initial loading doses in the first week on days 1, 3, and 5 followed by weekly dosing. Patients were eligible to remain on therapy until unacceptable toxicity or progression. Blood was collected pre- and post-treatment for analysis of peripheral immune cells.

**Results:**

Thirty patients were enrolled, 10 at 2 mg/kg and 20 at 3 mg/kg dose levels. Twenty-seven patients had DLBCL. AZD9150 was safe and well tolerated at both doses. Common drug-related adverse events included transaminitis, fatigue, and thrombocytopenia. The 3 mg/kg dose level is the recommended phase 2 dose. All responses were seen among DLBCL patients, including 2 complete responses with median duration of response 10.7 months and 2 partial responses. Peripheral blood cell analysis of three patients without a clinical response to therapy revealed a relative increase in proportion of macrophages, CD4+, and CD8+ T cells; this trend did not reach statistical significance.

**Conclusions:**

AZD9150 was well tolerated and demonstrated efficacy in a subset of heavily pretreated patients with DLBCL. Studies in combination with checkpoint immunotherapies are ongoing.

**Trial registration:**

Registered at ClinicalTrials.gov: NCT01563302. First submitted 2/13/2012.

**Electronic supplementary material:**

The online version of this article (10.1186/s40425-018-0436-5) contains supplementary material, which is available to authorized users.

## Background

The Janus kinases (JAKs) and signal transducer and activator of transcription (STAT) proteins are components of an intracellular cascade pathway that plays an important role in cancer. The JAK/STAT pathway was first recognized through its association with interferons α/γ and interleukins (IL) [1–3]. The downstream effect of cell-surface-level activation of the JAK/STAT pathway is a gene expression profile that results in enhanced cell survival, immune cell activation, and oncogenesis [4]. Increasing evidence supports the role of the JAK/STAT pathway in oncogenesis in both solid and hematologic malignancies [5, 6]. Significant interest has been directed toward understanding how the function or dysfunction of the JAK/STAT pathway contributes to oncogenic transformation and cancer cell survival [7–9]. Preclinical data suggest that aberrant activation of this pathway contributes to tumorigenesis [10] and to clonality and survival of cancer stem cells [11]. Targeting the JAK/STAT pathway can inhibit its downstream gene activation [12] and can suppress tumor growth [13, 14].

STAT3 is activated by phosphorylation of tyrosine residues by members of the JAK family recruited to the cytoplasmic portion of cell surface receptors, which are activated by growth factors and cytokines [2]. Phosphorylated STAT3 dimerizes and is translocated from the cytoplasm to the nucleus, where the dimer acts as a transcription factor for signals involved in cell proliferation, development, and differentiation and in inflammation and apoptosis. Constitutive activation of STAT3 is found in several types of human tumors [15, 16]. Hyperactivity of upstream growth factor receptors or non-receptor tyrosine kinases (e.g., Src, JAK, or Abl) or overexpression of stimulating ligands (e.g., epidermal growth factor receptor or IL-6) can produce a persistent STAT3 signal [17]. Constitutively active STAT3 has been shown to increase levels of tumor-associated signaling molecules such as survivin, Bcl-XL, cyclin D1/D2, C-Myc, Mcl-1, and vascular endothelial growth factor (VEGF), leading to increased cell proliferation, cell survival, angiogenesis, and oncogenesis [18–20].

STAT3 signaling also plays an important role in the regulation of the cancer stromal and immune cells of the tumor microenvironment. Ablation of STAT3 in the hematological compartment in a murine inducible knockout model improved the antitumor effect of neutrophils and natural killer cells, while depleting regulatory T-cells, suggesting that STAT3 signaling has a broad effect on multiple hematological compartments [21]. This enhancement of immune effectors resulting from STAT3 depletion could be mediated, in part, through enhancement of the antigen presentation capacity and co-stimulatory activation of dendritic cells. Multiple lines of evidence indicate that reversal of STAT3-mediated immunosuppression has the potential to augment the antitumor immune response [22]. Taken together, STAT3 is a particularly attractive cancer target as it not only regulates the expression of many genes that contribute directly to the survival and proliferation of tumor cells, but also supports immune-suppressive stromal cells within the tumor microenvironment, promoting tumor immune evasion, angiogenesis, and metastasis [23].

AZD9150 (ISIS 481464) is a 16-nucleotide next generation chemistry antisense oligonucleotide [24] designed to target and indirectly downregulate expression of human STAT3 protein by downregulating STAT3 mRNA. Preclinical activity has been shown in cell line and PDX lymphoma xenograft models, and initial single-agent studies of AZD9150 demonstrate its efficacy and clinical safety in patients with refractory lymphoma and lung cancer [25]. Here, we present the not previously reported results of a phase Ib expansion of the clinical trial of AZD9150 in patients with refractory/relapsed lymphoma. The primary goal of this expansion cohort was to assess safety, with exploratory endpoints investigating efficacy and immune cell changes among patients.

## Methods

### Patients

Eligible patients in the expansion cohort had a histologically confirmed lymphoma that had relapsed or became refractory after administration of at least 1 line of therapy and for which no standard therapy existed. Other eligibility requirements included age ≥ 18 years at enrollment, measurable disease per RESIST 1.1 criteria, Eastern Cooperative Oncology Group (ECOG) performance status ≤2, and life expectancy of at least 12 weeks. Willingness to provide pre- and post-treatment tumor tissue specimens was also required. Exclusion criteria included baseline cytopenias, significant cardiovascular disease, hepatic or renal dysfunction, known brain metastases, or prior concurrent malignancy in the past 3 years. Patients receiving ongoing anticoagulant therapy were also excluded. The clinical trial was reviewed and approved by the institutional IRB and compliant with human subject ethics guidelines. Informed consent was obtained from all patients prior to study enrollment. The trial was registered at www.clinicaltrials.gov as NCT01563302.

### Study design

The study was a phase I/Ib, multicenter, open-label study with a 3 + 3 dose-escalation design and a pre-planned dose expansion cohort. All patients received AZD9150, an ASO STAT3 inhibitor, as a single agent. In cycle 0, a loading regimen of AZD9150 was delivered intravenously at a given dose level with administration on Days 1, 3, and 5. Subsequently, in cycles 1 and beyond, a maintenance dose was administered weekly until disease progression, unacceptable toxicity, or patient discontinuation for any reason. The initial cohort of patients received 2 mg/kg AZD9150. Escalation of dose in this cohort was allowed based on initial toxicity and pharmacokinetic data from the dose escalation cohort. The decision to increase the dose to 3 mg/kg was made on the basis of safety data collected during cycle 0 (1 week) and cycle 1 (3 weeks). The decision not to pursue a higher dose was based on thrombocytopenia observed during dose escalation.

All patients were monitored clinically and with weekly blood tests to characterize the safety of AZD9150 and to assess preliminary evidence of clinical activity. Imaging was performed with CT or MRI of measurable sites and restaging imaging occurred every 8 weeks within a 7-day window. The recommended phase 2 dosing was selected on the basis of the toxicities observed in the first 28 days of dosing and preliminary evidence of clinical activity. Patients were enrolled in the expansion cohort with a goal of approximately 25 evaluable patients, defined as patients who completed the first restaging scan and underwent both pre- and post-treatment biopsies.

### Endpoints

The primary endpoint of the expansion cohort was to evaluate the safety and determine the recommended Phase 2 dose (RP2D) in advanced lymphoma. Secondary objectives included measuring clinical activity of AZD9150. This included objective response rate, defined as the percentage of patients with complete response (CR) or partial response (PR) as the best response. For this purpose, disease burden was evaluated according to the Response Evaluation Criteria in Solid Tumors (RECIST) version 1.1 for solid tumors or the International Workshop Response Criteria (IWRC) for Non-Hodgkin’s Lymphoma. Responses were confirmed using IWRC [ref]. Additional secondary efficacy endpoints included clinical benefit rate, defined as the percentage of patients with CR, PR, or stable disease (SD) for more than 4 months as the best response; progression-free survival duration; and duration of response for responding patients, defined as the time from the date of the first objective status assessment of CR or PR to the date of disease progression. Results of changes in myeloid cell populations of peripheral blood mononuclear cells (PBMCs) were presented as absolute changes and percentage changes from baseline over time after study drug administration.

Safety assessments, including clinical assessment, toxicity monitoring, and blood testing were conducted for all patients in the expansion arm on a weekly basis. At any time, if 33% or more of the participants experienced a dose-limiting toxicity within the monitoring window, enrollment was held and resumed at the next lowest dose level. All patients who enrolled were accounted for in a patient disposition analysis. All patients who received any part of a dose of the study treatment were included in the full analysis population. This population was the basis for all data on demographic and baseline disease characteristics and clinical activity.

### Adverse events

Adverse events (AEs) occurring from study day 1 until 4 weeks after the last dose of AZD9150 were recorded and graded using the adult National Cancer Institute Common Terminology Criteria for Adverse Events (CTCAE, version 4.0). AE terms were coded using the most current version of the Medical Dictionary for Regulatory Activities (MedDRA). The incidence of all treatment-emergent AEs was summarized by system, organ, class, and preferred term. Investigators categorized AEs as being at least possibly related to AZD9150 or unrelated. AEs reported as being related to AZD9150 were classified as treatment-related AEs.

### Tissue procurement and NGS

For pre-treatment biopsy samples either fresh or archival tissue was used, however tissue had to have been obtained within 28 days of first dose. The post dose biopsy timing was determined on a per patient basis based on response. All tissue was obtained via needle biopsy conducted by an interventional radiologist and at least 3 cores were obtained at each time point. All samples were fixed in formalin and paraffin embedded and sent to the sponsor for analysis.

Genomic analysis was performed using a clinical NGS-based assay (FoundationOne™Heme, Foundation Medicine Inc., Cambridge, MA) as previously described [26]. The sequencing method was validated on hybridization captured, adaptor ligation-based libraries using DNA extracted from ten formalin-fixed paraffin-embedded (FFPE) sections cut at 5 μm [26]. Adaptor-ligated sequencing libraries were captured by solution hybridization with two custom bait-sets targeting 374 cancer-related genes, 31 genes frequently rearranged by DNA-seq and 265 genes frequently rearranged by RNA-seq. This method was validated also for the detection of copy number alterations, including amplification and deletions, by a statistical model normalized to exonic coverage and allele frequencies.

### Peripheral blood procurement and analysis

PBMCs were collected from 4 patients both before treatment and at the completion of each cycle of therapy. Each sample was separated by gradient centrifugation, and the mononuclear cells were collected and processed for flow cytometry. Following density gradient separation, samples were fixed using the Foxp3/Transcription Factor Staining Buffer Set (eBioscience) and then stained with up to 16 antibodies at a time from Biolegend, BD Biosciences, eBioscience, and Life Technologies. Flow cytometry data was collected on a custom 5-laser, 18-color BD LSR II cytometer and analyzed using FlowJo Version 7.6.5 (Treestar).

## Results

### Patient characteristics

Thirty-three lymphoma patients were enrolled at 9 sites in the United States. Of these patients, 30 received at least 1 infusion of AZD9150 between February 27, 2012 and November 20, 2014 (Table 1). Data for all patients is current as of cutoff of July 2016. All patients had histologically confirmed lymphoma, 27 patients had DLBCL, 2 had follicular lymphoma, and 1 had Hodgkins lymphoma. The median age of participants was 69 years, and most (93%) had an ECOG performance status of 1 or better. The majority of patients (80%) had stage III or IV disease. All patients had received prior systemic therapies for their cancer. The median number of prior treatment regimens was 4 (range, 1–9). About a third of the patients had received prior radiation therapy, and 20% had received prior surgery for their disease. Approximately 27% of patients had a previous cancer diagnosis.

**Table 1:** Patient demographics and baseline clinical characteristics by dose level

Characteristic	2 mg/kg (*n =* 10)	3 mg/kg (*n =* 20)
Median age, y (range)	69 (23–83)	65 (22–81)
Male, n (%)	8 (80)	10 (50)
White, n (%)	8 (80)	20 (100)
ECOG performance status, n (%)		
0	4 (40)	3 (15)
1	6 (60)	15 (75)
2		2 (10)
Disease stage, n (%)		
I	0	2 (10)
II	1 (10)	3 (15)
III	4 (40)	3 (15)
IV	5 (50)	12 (60)
Prior treatment with radiation therapy, n (%)	3 (30)	6 (30)
Prior treatment with systemic therapy, n (%)	10 (100)	20 (100)
Median number of prior regimens (range)	4.5 (1–8)	3.5 (1–9)
Prior treatment with surgery, n (%)	3 (30)	3 (15)
History of cancer(s) other than current cancer, n (%)	5 (50)	3 (15)

### Treatment duration and toxicities

Patients received a median of 2 cycles of AZD9150 (range, 1–21). Five patients received 4 or more cycles of therapy. The most common reason for discontinuing treatment was disease progression (27 patients, 75%). Treatment was terminated among the remaining patients for the following reasons: 5 (14%) voluntarily withdrew consent, 1 (3%) withdrew at recommendation of investigator, 1 (3%) due to ineligibility, and 2 (6%) for other reasons. No patient withdrew from the study because of toxicities related to AZD9150. The pattern and frequency of AEs was not significantly different between dose levels.

Of the 30 patients treated with at least 1 dose of AZD9150, 29 experienced at least 1 AE (Table 2). The most common drug-related AEs in patients were transaminitis (alanine transaminase or aspartate transaminase elevation, 40%), fatigue (37%), thrombocytopenia (30%), nausea (20%), and anemia, hypomagnesemia, and peripheral edema (each 17%). The majority of reported toxicities were grade 1 or 2, however higher-grade thrombocytopenia was more common with 3 grade 3 and 2 grade 4 events. Eighteen patients (60%) experienced at least one grade 3 or higher AE and 5 (17%) had events that were at least possibly therapy-related. One patient died from acute respiratory failure while participating in the study; this death resulted from underlying comorbidities and was not related to the patient’s participation in the study or to the study drug.

**Table 2:** Adverse events experienced by at least 10% of patients by severity

AE, *n* (%)	Total (*N =* 30)	Grade 1	Grade 2	Grades 3 and 4
Any AE, highest grade experienced^a^	29 (97)	2 (7)	8 (27)	18 (60)
ALT elevation	12 (40)	5 (17)	5 (17)	2 (7)
AST elevation	12 (40)	7 (23)	4 (13)	1 (3)
Fatigue	11 (37)	4 (13)	5 (17)	2 (7)
Thrombocytopenia	9 (30)	2 (7)	2 (7)	5 (17)
Nausea	6 (20)	2 (7)	3 (10)	1 (3)
Anemia	5 (17)	2 (7)	2 (7)	1 (3)
Hypomagnesemia	5 (17)	5 (17)	0	0
Peripheral edema	5 (17)	3 (10)	2 (7)	0
Alkaline phosphatase elevation	4 (13)	3 (10)	1 (3)	0
Dysphagia	4 (13)	3 (10)	0	1 (3)
Dyspnea	4 (13)	2 (7)	2 (7)	0
Hypercalcemia	4 (13)	1 (3)	2 (7)	1 (3)
Hypokalemia	4 (13)	3 (10)	0	1 (3)
Hyponatremia	4 (13)	3 (10)	0	1 (3)
Neutropenia	4 (13)	1 (3)	0	3 (10)
Vomiting	4 (13)	4 (13)	0	0
Abdominal pain	3 (10)	1 (3)	2 (7)	0
Anorexia	3 (10)	1 (3)	2 (7)	0
Asthenia	3 (10)	1 (3)	1 (3)	1 (3)
Constipation	3 (10)	3 (10)	0	0
Creatinine elevated	3 (10)	2 (7)	1 (3)	0
Diarrhea	3 (10)	3 (10)	0	0
Upper respiratory tract infection	3 (10)	1 (3)	2 (7)	0
Urinary tract infection	3 (10)	1 (3)	2 (7)	0

*Abbreviations*: *AE* adverse event, *AST* aspartate transaminase, *ALT* alanine transaminase

^a^Includes one patient who died of acute respiratory failure (Grade 5 AE) unrelated to the study medication while on trial

### Antitumor activity

**Fig. 1:**
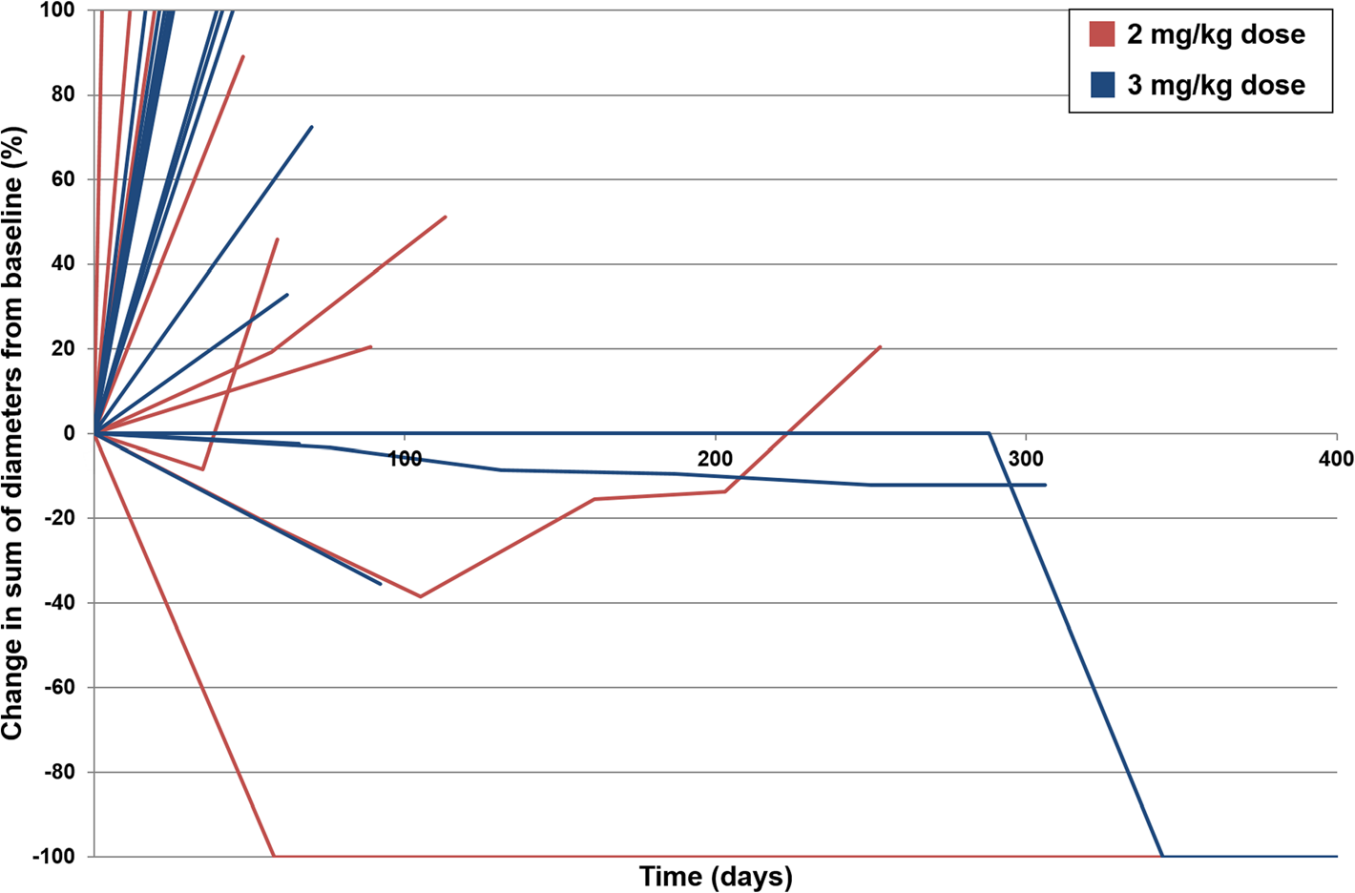
Spider plot of percentage change in tumor size during treatment

**Fig. 2:**
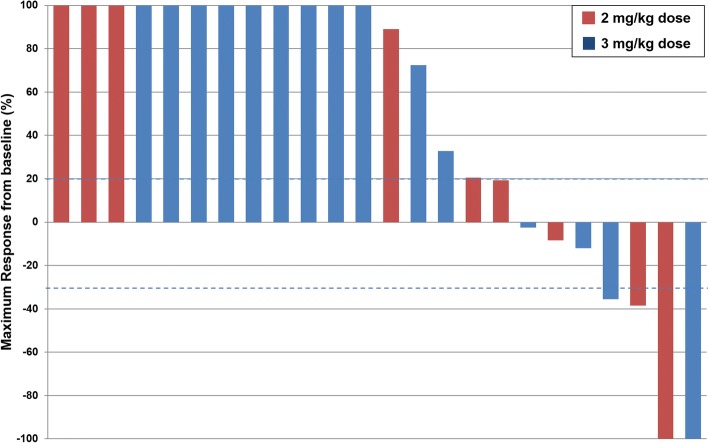
Waterfall plot of best responses seen in 24 evaluable patients. Blue dotted lines are reference for partial response (− 30%) and progressive disease (+ 20%)

All 30 patients who received at least 1 dose of AZD9150 were assessed for response to treatment. The percent change in tumor size during treatment is presented as a spider plot in Fig. 1. All responses were observed among patients with DLBCL. Two (7%) patients had a CR to therapy: 1 each at the 2 mg/kg and 3 mg/kg dose levels. The median duration of response at data cutoff was 10.7 months; however, one response was ongoing at last follow-up. Two (7%) patients had a PR to therapy at initial restaging. One progressed clinically and the other progressed on subsequent restaging after 5 months of therapy. One (3%) patient had SD as the best response. A total of 4 patients, all with DLBCL, had PR, CR, or SD for at least 4 months, for a clinical benefit rate of 13%. There was no clear difference in progression-free survival or objective response rate between the 2 dose levels (Fig. 2).

### Mutational analysis of responder

A pretreatment biopsy specimen was obtained for the patient who demonstrated a persistent complete response to therapy. Genomic analysis identified the following mutations (percent-reads, coverage): a known somatic short-variant, CD79B_c.587A>C_p.Y196S (0.34,604), a likely somatic short-variant, BCL10_c.657_699delTGAGATGTTTCTTCCCTTAAGATCACGTACTGTTTCACGACAA_p.E220fs*1+ (0.23,474), and homozygous deletions of CDKN2A and CDKN2B in 5 of 5 exons. Other mutations of interest were identified in CCND3, FOXP1, IRF4, PCLO, and a rearrangement of BCL6/FOXP1 (full list in appendix 1).

### Changes in PBMCs

**Fig. 3:**
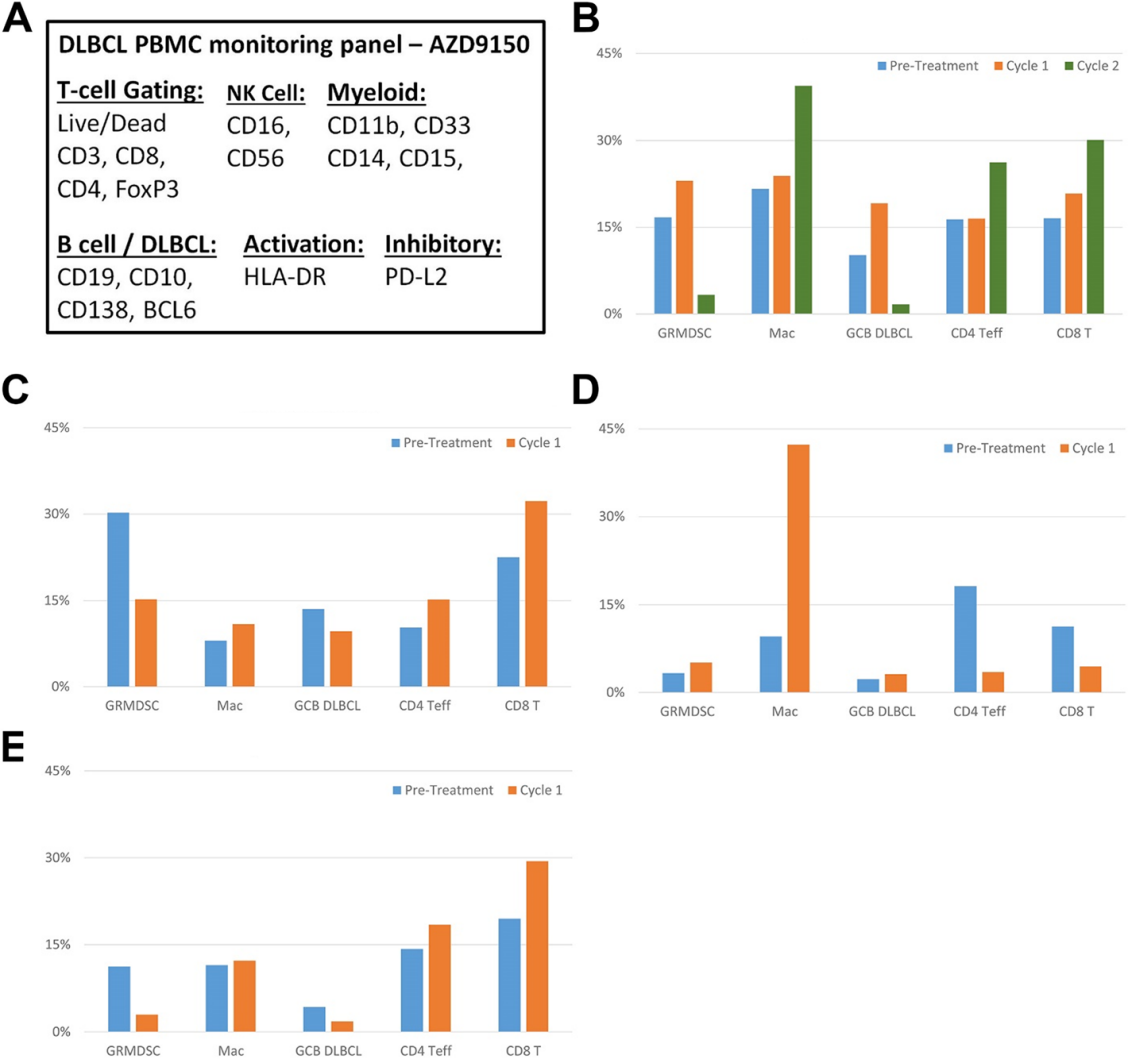
Changes in patient PBMC profiles following AZD9150 therapy (PBMC subpopulations with frequencies of less than 2% are not shown). **a** Surface markers analyzed. **b**-**e** Patient PBMC populations before (blue) and after 1 (orange) or 2 (green) cycles of therapy

Peripheral blood was collected from 4 patients prior to and on treatment including 1 at 2 mg/kg and 3 at 3 mg/kg dose levels. Three of the patients completed Cycle 1 and then ceased treatment because of disease progression, and the remaining patient completed Cycles 1 and 2 (Fig. 3a). The PBMC populations of the latter patient showed clear evidence of peripheral immunomodulation by AZD9150, with 5-fold downregulation of granulocytic myeloid-derived suppressor cells (Gr-MDSCs; CD11b^+^CD33^+^CD15^+^HLA-DR^−/low^) and a greater than 1.5-fold upregulation of macrophages (CD11b^+^CD33^−^), CD4^+^ effector T cells, and CD8^+^ T cells (Fig. 3b). In addition, this patient’s circulating DLBCL tumor cell frequency decreased more than 6-fold, from 10.2 to 1.7% of PBMCs. Of the 3 patients who received a single cycle of therapy, the PBMC profiles of 2 resembled that of the previously described patient (Fig. 3c), whereas the third showed increases in Gr-MDSCs and circulating tumor cell frequency and decreases in effector CD4^+^ and CD8^+^ T- cell frequencies (Fig. 3d). This patient’s PBMCs became dominated by an expanded macrophage population after the first cycle of therapy.

## Discussion

In this trial of the next-generation STAT3 ASO, AZD9150 in patients with DLBCL, the drug was well tolerated at doses of 2 or 3 mg/kg weekly. No significant AEs associated with AZD9150 led to unacceptable toxicity or early discontinuation of therapy in the study population. We observed a clinical benefit in 13% of the study population of patients with heavily pretreated DLBCL. Among both patients with a CR, the response was durable, lasting nearly 11 months in 1 patient and is currently ongoing in the other. Corollary PBMC analysis shows changes in relevant T cell populations with therapy.

Activated STAT3 plays an established role in cancer cell survival, and suppressed levels of phosphorylated STAT3 have shown to correlate with better patient survival in lymphoma. We have previously reported the efficient uptake and STAT3 knockdown capacity of AZD9150 in patient-derived tumor explant models of lymphoma [25]. The results of the current study demonstrate that this next-generation ASO-mediated reduction of STAT3 mRNA levels in humans is both a feasible and an effective strategy for treating advanced-stage DLBCL. An important finding is that this therapy was well tolerated, confirming our early phase 1 results [25]. The majority of the patients had undergone several lines of therapy—a median of 4—for their current cancer. Notably, over one quarter of the patients had a prior diagnosis of cancer, and may in fact have received even more systemic therapies. Patients withdrew from the study in most cases because of clinical deterioration unrelated to AZD9150 treatment or because of disease progression. Overall, our results suggest that AZD9150 therapy would likely be well tolerated and have meaningful clinical activity at a dose of 3 mg/kg in the broader population of patients with DLBCL.

Among activated B-cell (ABC) type DLBCL there is evidence of increased JAK-STAT and pSTAT3 activation [27]. Comprehensive genomic analysis was performed in the pretreatment biopsy specimen of a patient who experienced a complete response of therapy. Multiple known pathogenic mutations were identified including in CD79B, ERBB2, RET, and homozygous deletions in tumor suppressors CDKN2A/B (Additional file 1: Table S1). CD79B is a subunit of the B-cell-receptor (BCR) and has been implicated as an oncogenic driver, primarily in activated B-cell (ABC) DLBCL, through mutations that lead to a chronically active state [28]. In this patient, we hypothesize that combination of multiple activating mutations and loss of tumor suppressors contributed to tumor progression through over-activation of the JAK-STAT pathway; and was effectively suppressed through direct targeting of STAT3. Given the recognized over-activation of JAK-STAT signaling in advanced ABC-type DLBCL, the mechanism of anti-tumor effect of STAT targeting therapy should be further investigated in future studies. Additionally, NK/T-cell lymphomas can be driven by activation of STAT3 [29] and may be rationale diseases to target with STAT inhibition [30, 31].

In the peripheral blood, we observed increases in the both lymphoid and myeloid cell populations. In three of four patients, this resulted in a favorable shift with increased CTLs and decreased MDSCs. While the numbers of patients analyzed was too small for correlative analysis, the consistent pattern of Gr-MDSC and DLBCL cell downregulation in the context of macrophage and CD4^+^ and CD8^+^ effector T-cell upregulation suggests a possible pharmacologic biomarker signature of response to STAT3 ASO treatment. These findings are consistent with recently presented data demonstrating the ability of a murine specific STAT3 ASO to suppress CD163 and Arginase in macrophages in the tumor microenvironment of syngeneic tumor models [32]. The role of regulatory T cells and response to therapy is potentially relevant, however we did not observe a clear trend in our data (Additional file 2: Figure S1). In follicular lymphoma, there is evidence that TGF-β induces CD70 on T-cells leading to an exhausted phenotype that is associated with worse patient outcomes [33]. The ability to reduce immunosuppressive cells in the microenvironment may synergize with therapies that enhance cytotoxic lymphocytes. One limitation of this study is a lack of PBMC data from patients with persistent clinical responses and this analysis would be of certain value in future studies.

Emerging evidence shows that many T-cell immune responses are limited by the development of a suppressive myeloid phenotype [34, 35]. Adding therapies that target MDSCs may improve the efficacy of existing immunotherapies. For instance, tumor-induced VEGF acts through JAK/STAT signaling to induce MDSCs with immunosuppressive functionality [36]. Interestingly, genetic inhibition of STAT3 function has been shown to reduce the immunosuppressive capability of MDSCs, even in the setting of co-stimulatory signaling that results in expansion of the MDSC population [37]. Taken together, this suggests further efforts are need to better characterize myeloid subpopulations before and as a consequence of therapy.

## Conclusions

In conclusion, the results of this clinical trial provide evidence that AZD9150, a next-generation ASO inhibitor of STAT3 mRNA, is safe and appears to benefit some patients with heavily pretreated DLBCL. Given the clear evidence of accumulation of ASO and suppression of STAT3 in the tumor microenvironment [25], it is likely that AZD9150 is exerting a positive immunomodulatory effect and clinically meaningful antitumor activity. Trials to combine this agent with checkpoint-targeting immunotherapies are in progress.

## Additional files

Additional file 1: **Table S1.** Genomic analysis of pre-treatment tumor in complete responder with DLBCL. **Table S2.** Peripheral blood cell counts of patients reported in Fig. 3 on days of peripheral blood mononuclear cell analysis with fold-change in absolute number. (DOCX 16 kb)

Additional file 2: **Figure S1.** Relative percentage of FoxP3+CD4+T cells before and after treatment.. (PNG 43 kb)

## Abbreviations

ABC: Activated B-cell
AE: Adverse event
ASO: Anti-sense oligonucleotide
BCR: B-cell receptor
CR: Complete response
CT: Computed tomography
CTCAE: Common terminology criteria for adverse events
DLBCL: Diffuse large B-cell lymphoma
ECOG: Eastern cooperative oncology group
FFPE: Formalin-fixed paraffin-embedded
IL: Interleukin
JAK: Janus kinase
MDSC: Myeloid-derived suppressor cells
MedDRA: Medical dictionary for regulatory activities
MRI: Magnetic resonance imaging
mRNA: Messenger ribonucleic acid
MTD: Max tolerated dose
NGS: Next-generation sequencing
PBMC: Peripheral blood mononuclear cell
PR: Partial response
RECIST: Response evaluation criteria in solid tumors
RNA: Ribonucleic acid
RP2D: Recommended phase 2 dose
SD: Stable disease
STAT: Signal transduction and activation of transcription
VEGF: Vascular endothelial growth factor
