# Supplementary material for: STAT3 antisense oligonucleotide AZD9150 in a subset of patients with heavily pretreated lymphoma: results of a phase 1b trial
Source: J Immunother Cancer. 2018 Nov 16;6:119. doi: 10.1186/s40425-018-0436-5 (PMC6240242; doi:10.1186/s40425-018-0436-5)
Supplement: Supplementary file 1 — Table S1. Genomic analysis of pre-treatment tumor in complete responder with DLBCL. Table S2. Peripheral blood cell counts of patients reported in Fig. 3 on days of peripheral blood mononuclear cell analysis with fold-change in absolute number. (DOCX 16 kb) [file 40425_2018_436_MOESM1_ESM.docx]

| Disease | Tumor Nuclei (%) | Median Exon Coverage | Somatic Variants (% reads, coverage) | Homozygous Deletions (exons) | Rearrangements (supporting reads) |
| --- | --- | --- | --- | --- | --- |
| DLBCL | 30 | 524 | BCL10_c.657_699delTGAGATGTTTCTTCCCTTAAGATCACGT  ACTGTTTCACGACAA_p.E220fs*1+(0.23,474)  CCND3_c.595C>T_p.Q199*(0.3,571)  CD79B_c.587A>C_p.Y196S(0.34,604)  CIITA_c.3004G>A_p.E1002K(0.33,724)  EP300_c.6798_6800delGCA_p.Q2266_Q2267>Q(0.49,501)  ERBB2_c.140G>A_p.R47H(0.46,415)  FOXP1_c.1709A>G_p.N570S(0.49,528)  IRF4_c.1022C>T_p.A341V(0.49,523)  MAF_c.433g>A_p.G145S(0.59,49)  PCLO_c.6492G>C_p.L2164F(0.47,603)  PCLO_c.6398C>A_p.T2133N(0.51,590)  PIK3C2G_c.1744C>G_p.L582V(0.33,463)  PRDM1_c.1906T>C_p.C636R(0.47,393)  RET_c.3307C>T_p.P1103S(0.52,646) | CDKN2A (5 of 5)  CDKN2B (5 of 5) | BCL6_FOXP1 (126) |

Table S1: Genomic analysis of pre-treatment tumor in complete responder with DLBCL.

| **Patient** | **Absolute Lymphocyte Count (fold change from baseline)** | | | **Absolute Monocyte Count (fold change from baseline)** | | |
| --- | --- | --- | --- | --- | --- | --- |
|  | Pre (C0D1) | Post C1 (C2D1) | Post C2 (C3D1) | Pre (C0D1) | Post C1 (C2D1) | Post C2 (C3D1) |
| B | 610 (1) | 950 (1.6) | 1480 (2.4) | 430 (1) | 830 (1.9) | 750 (1.7) |
| C | 550 (1) | 1850 (3.4) |  | 1220 (1) | 1110 (0.9) |  |
| D | 510 (1) | 400 (0.8) |  | 1100 (1) | 260 (0.2) |  |
| E | 1240 (1) | 1850 (1.5) |  | 400 (1) | 400 (1) |  |

Table S2: Peripheral blood cell counts of patients reported in figure 3 on days of peripheral blood mononuclear cell analysis with fold-change in absolute number.
