# Supplementary figures and images for: STAT3 antisense oligonucleotide AZD9150 in a subset of patients with heavily pretreated lymphoma: results of a phase 1b trial
Source: J Immunother Cancer. 2018 Nov 16;6:119. doi: 10.1186/s40425-018-0436-5 (PMC6240242; doi:10.1186/s40425-018-0436-5)

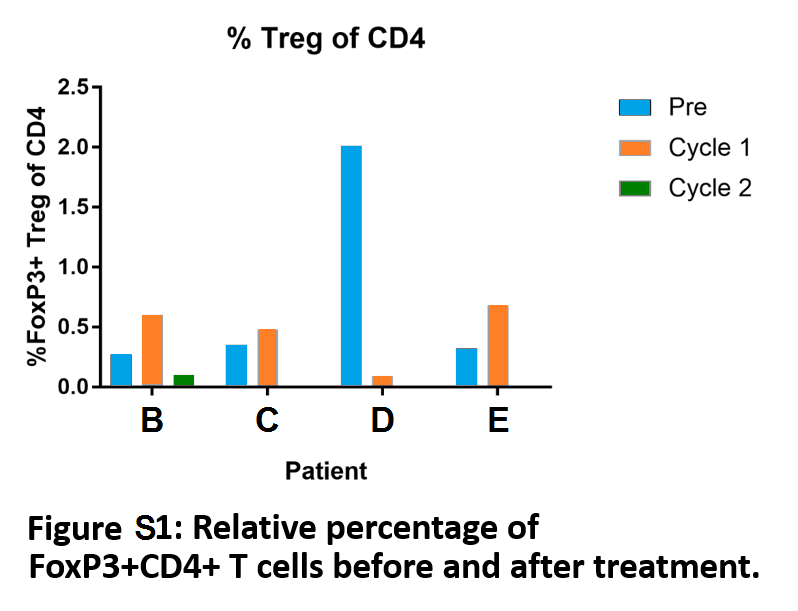

Supplement: Supplementary file 2 — Figure S1. Relative percentage of FoxP3+CD4+T cells before and after treatment.. (PNG 43 kb) [file 40425_2018_436_MOESM2_ESM.png]
